# Relationship between intraocular pressure lowering effect and chemical structure of imidazo[1,2-a]benzimidazole and pyrimido[1,2-a]benzimidazole derivatives

**DOI:** 10.1016/j.dib.2018.02.067

**Published:** 2018-03-06

**Authors:** Pavel Vassiliev, Igor Iezhitsa, Renu Agarwal, Adrian Julian Marcus, Alexander Spasov, Olga Zhukovskaya, Vera Anisimova

**Affiliations:** a Volgograd State Medical University, Research Institute of Pharmacology, Volgograd, Russia; b Centre for Neuroscience Research, Faculty of Medicine, Universiti Teknologi MARA, Malaysia; c Southern Federal University, Research Institute of Physical and Organic Chemistry, Rostov-on-Don, Russia

**Keywords:** QSAR, *In silico* drug design, Intraocular pressure;, Intraocular pressure lowering activity, Hypotensive activity;, Imidazo[1,2-a]benzimidazoles, Pyrimido[1,2-a]benzimidazoles, Pharmacophore analysis, Artificial neural network modeling

## Abstract

This article contains data that relate to the study carried out in the work of Marcus et al. (2018) [1]. Data represent an information about pharmacophore analysis of imidazo[1,2-a]benzimidazole and pyrimido[1,2-a]benzimidazole derivatives and results of construction of the relationship between intraocular pressure (IOP) lowering activity and hypotensive activity of imidazo[1,2-a]benzimidazole and pyrimido[1,2-a]benzimidazole derivatives using a multilayer perceptron artificial neural network. In particular, they include the ones listed in this article: 1) table of all pharmacophores of imidazo[1,2-a]benzimidazole and pyrimido[1,2-a]benzimidazole derivatives that showed IOP lowering activity; 2) table of all pharmacophores of the compounds that showed absence of IOP lowering activity; 3) table of initial data for artificial neural network analysis of relationship between IOP activity and hypotensive activity of this chemical series; 4) graphical representation of the best neural network model of this dependence; 5) original txt-file of results of pharmacophore analysis; 6) xls-file of initial data for neural network modeling; 7) original stw-file of results of neural network modeling; 8) original xml-file of the best neural network model of dependence between IOP lowering activity and hypotensive activity of these azole derivatives. The data may be useful for researchers interested in designing new drug substances and will contribute to understanding of the mechanisms of IOP lowering activity.

**Specifications Table**

**Table:** 

*Subject area*	Medicine
*More specific subject area*	Pharmacology, QSAR, *In silico* drug design
*Type of data*	Tables, figure, *.txt, *.xls, *.stw and *.xml files
*How data was acquired*	This study was done in ocular normotensive rats and rebound tonometry (Tonolab, Icare Finland) was used to estimate intraocular pressure (IOP). Pharmacophore analysis was carried out using IT Microcosm package (Russian Federation). Neural network modeling was performed using Statistica 6.0 package (StatSoft Inc., USA).
*Data format*	Analyzed
*Experimental factors*	This data is supplementary to article [1]. A total of 27 new compounds were synthesized as described previously and tested for IOP lowering effect in rats. These compounds included twenty 9H-imidazo[1,2-a]benzimidazoles, four 10H-pyrimido[1,2-a]benzimidazoles, two 1H-pyrimido[1,2-a]benzimidazoles and one 1H-imidazo[1,2-a]benzimidazole [2], [3], [4], [5], [6], [7], [8], [9], [10], [11].
*Experimental features*	All compounds were topically applied as a single drop, unilaterally, at 3 different concentrations (0.1%, 0.2% and 0.4%). The contralateral eye was instilled with vehicle and served as control. The IOP reduction was measured up to 6 hours. In pharmacophore analysis, the chemical structure of the compounds was represented in the form of descriptors of the QL language.
*Data source location*	Volgograd State Medical University, Research Institute of Pharmacology, Volgograd, Russia
*Data accessibility*	The data are available in this article and in appended files.
*Related research article*	This data is supplementary to article [1].

**Value of the Data**

• The data include the results of pharmacophore analysis of IOP lowering activity of imidazo[1,2-a]benzimidazole and pyrimido[1,2-a]benzimidazole derivatives and may be useful for researchers interested in designing new drug substances.
• IOP lowering activity and pharmacophore list will help other researchers in investigating new drugs.
• These data can be compared with the data of pharmacophore analysis performed by other researchers and this will facilitate international collaborations in the field of drug development.
• The results of analysis of the relationship between IOP activity and hypotensive activity of imidazo[1,2-a]benzimidazole and pyrimido[1,2-a]benzimidazole derivatives with the help of artificial neural networks will contribute to understanding of the mechanisms of IOP lowering activity.

## 1 Data

**Fig. 1:**
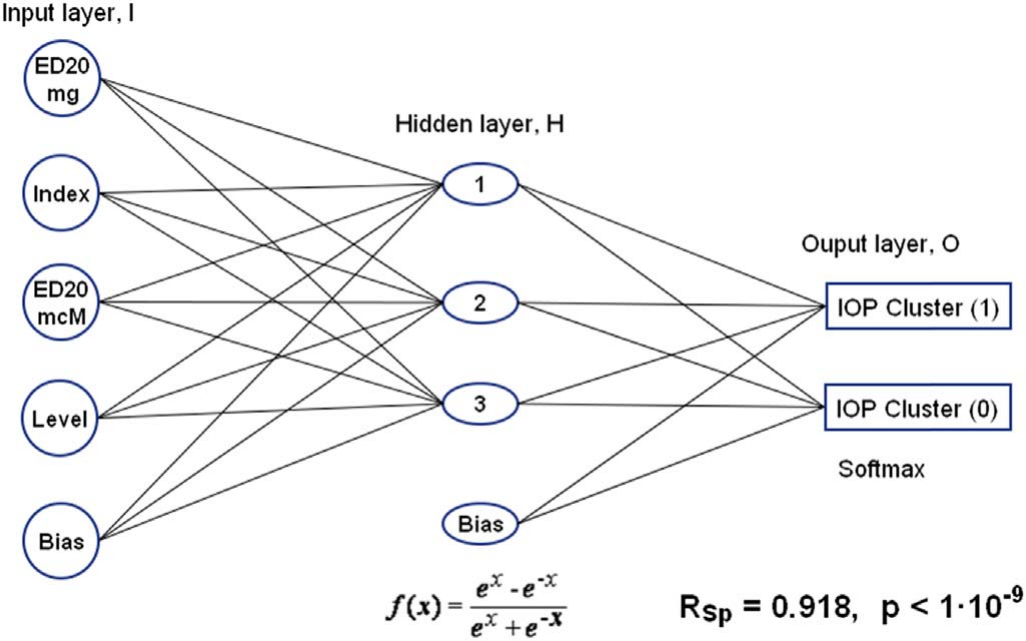
The architecture of a multilayer perceptron artificial neural network describing the relationship between IOP lowering activity and hypotensive activity of imidazo[1,2-a]benzimidazole and pyrimido[1,2-a]benzimidazole derivatives.

Benzimidazoles are heterocyclic compounds that are known for numerous therapeutic effects and are recognized as important pharmacophore in drug discovery [1], [2], [3], [4], [5], [6], [7], [8], [9], [10], [11], [12]. Over the past years, several benzimidazole derivatives have been synthesized and their pharmacological activities investigated. Some benzimidazoles have previously shown hypotensive activity in normotensive rats [1], [3], [4]. We hypothesized that the IOP lowering activity is linked with hypotensive activity; perhaps this dependence is non-linear and discrete. This data is supplementary to [1] presenting the effects of benzimidazole-based compounds on IOP of ocular normotensive rats. These compounds included twenty 9H-imidazo[1,2-a]benzimidazoles, four 10H-pyrimido[1,2-a]benzimidazoles, two 1H-pyrimido[1,2-a]benzimidazoles and one 1H-imidazo[1,2-a]benzimidazole [6], [7], [8], [9], [10], [11]. In present article, data combine the results of pharmacophore and neural network analysis. These are the pharmacophores of the compounds showing presence (Table 1) and the absence (Table 2) of the IOP lowering activity (also the file "Pharmacophores IOP High 5e-2.txt" in appendix) and the neural network model of the relationship of IOP lowering activity and hypotensive activity for these derivatives (Table 3, Fig. 1 and also the files "Data for Networks IOPout HTin.xls", "Networks IOPoutHTin.stw", "SANN_PMML_Code_Data for Networks IOPout_HTin-34.xml" in appendix).

**Table 1:** Pharmacophores of imidazo[1,2-a]benzimidazole and pyrimido[1,2-a]benzimidazole derivatives showing presence of intraocular pressure lowering activity.

SD1	LD	SD2	BD	*P*_a_	*P*_i_	Pr
	1			0.2245	0.2164	3.79E-02
	−1			0.2473	0.2220	1.58E-02
			…1	0.3113	0.2833	1.41E-02
			p..1	0.0310	0.0222	4.73E-02
−N<	1			0.0600	0.0573	4.69E-02
−N<	2			0.0682	0.0676	4.72E-02
>N+=	1			0.0025	0.0003	2.86E-02
>N+=	2			0.0031	0.0003	1.03E-02
>N+=	3			0.0025	0.0003	2.86E-02
>S	2			0.0031	0.0003	1.03E-02
−C(Ar)<	−1			0.0675	0.0673	4.75E-02
Cyc06	−1			0.0269	0.0213	3.55E-02
>N+=		−C(Ar)<		0.0094	0.0005	4.95E-04
>O		−C(Ar)<		0.0247	0.0163	3.82E-02
=NH			p..1	0.0025	0.0003	2.81E-02
−N<			…1	0.0532	0.0449	2.41E-02
−N<			.A.1	0.0595	0.0568	4.74E-02
−N=			…0	0.0057	0.0024	3.81E-02
>N+=			…0	0.0025	0.0003	2.81E-02
>N+=			.a.0	0.0032	0.0003	1.01E-02
>N+=			.A.1	0.0025	0.0003	2.81E-02
>O			.A.1	0.0120	0.0070	2.60E-02
>S			…0	0.0025	0.0003	2.81E-02
>S			.a.0	0.0032	0.0003	1.01E-02
>C=			…1	0.0101	0.0062	4.37E-02
−C(Ar)<			…1	0.0925	0.0923	4.14E-02
Cyc06			…0	0.0304	0.0227	2.02E-02
CycAr06			…1	0.0754	0.0725	4.28E-02
	1		…1	0.1049	0.0951	4.20E-02
	−1		…0	0.0550	0.0429	3.24E-02
	−1		…1	0.1816	0.1750	4.09E-02
>N+=	2	−C(Ar)<		0.0041	0.0005	3.74E-02
Cyc06	−1		…0	0.0245	0.0204	4.77E-02
>N+=		−C(Ar)<	.A.1	0.0043	0.0005	3.54E-02

SD – structure descriptor; LD – length descriptor; BD – bond descriptor; *P*_a_ – frequency for a class of active compounds; *P*_i_ – frequency for a class of inactive compounds; Pr – significance in hypergeometric test [10].

**Table 2:** Pharmacophores of imidazo[1,2-a]benzimidazole and pyrimido[1,2-a]benzimidazole derivatives showing absence of intraocular pressure lowering activity.

SD1	LD	SD2	BD	*P*_a_	*P*_i_	Pr
	2			0.2204	0.2214	4.18E-02
	3			0.1438	0.1471	4.83E-02
	4			0.0833	0.0943	4.18E-02
			…0	0.2049	0.2133	3.83E-02
			.a.1	0.1725	0.1767	4.45E-02
			.A.1	0.2102	0.2200	3.66E-02
			pA.1	0.0040	0.0133	1.80E-02
−CH3	2			0.0044	0.0083	4.25E-02
>C(<)	1			0.0269	0.0323	4.09E-02
−C(Ar)<	5			0.0113	0.0156	4.91E-02
−N<		−C(Ar)<		0.1943	0.1966	4.11E-02
−OH		−C(Ar)<		0.0012	0.0089	1.04E-02
=O		−C(Ar)<		0.0059	0.0184	4.43E-03
>PH<		−C(Ar)<		0.0012	0.0110	2.89E-03
−Cl		−C(Ar)<		0.0012	0.0079	1.94E-02
−CH3		−C(Ar)<		0.0012	0.0068	3.57E-02
>C=		−C(Ar)<		0.0059	0.0184	4.43E-03
−C(Ar)<		CycAr06		0.1154	0.1264	3.66E-02
−N<			…0	0.0551	0.0582	4.72E-02
−OH			.A.1	0.0006	0.0046	1.26E-02
=O			pA.1	0.0019	0.0065	1.52E-02
−Cl			…0	0.0006	0.0076	3.99E-04
−Cl			.A.1	0.0006	0.0046	1.26E-02
>C=			.A.1	0.0019	0.0065	1.52E-02
>C(<)			…0	0.0709	0.0742	4.26E-02
−C(Ar)<			.a.1	0.0551	0.0574	4.95E-02
−C(Ar)<			.A.1	0.0861	0.0928	3.12E-02
−C(Ar)<			pA.1	0.0006	0.0046	1.26E-02
	1		…0	0.0575	0.0663	5.00E-02
	2		…0	0.0550	0.0647	4.64E-02
−C(Ar)<	−1	CycAr06		0.0919	0.1006	3.98E-02
>C(<)	1		…0	0.0245	0.0310	2.83E-02
−C(Ar)<	−1		…1	0.0616	0.0646	4.32E-02
=O		−C(Ar)<	pA.1	0.0011	0.0085	8.11E-03
−Cl		−C(Ar)<	.A.1	0.0011	0.0060	4.06E-02
>C=		−C(Ar)<	.A.1	0.0011	0.0085	8.11E-03
−C(Ar)<		CycAr06	…1	0.1002	0.1067	4.51E-02
−C(Ar)<		CycAr06	.A.1	0.0011	0.0075	1.56E-02
−C(Ar)<	−1	CycAr06	…1	0.0875	0.0982	3.30E-02

**Table 3:** Initial data for artificial neural network analysis of relationship between intraocular pressure lowering activity and hypotensive activity of imidazo[1,2-a]benzimidazole and pyrimido[1,2-a]benzimidazole derivatives.

Code	IOP Cluster	ED_20_ mg/kg	Index	ED_20_ mcM/kg	Level
RU 0185	0	6.60	2	12.54	1
RU 0238	0	22.40	1	51.92	1
RU 0239	0	19.00	1	43.85	1
RU 0243	0	11.70	1	25.47	1
RU 0244	0	15.00	1	32.51	1
RU 0247	1	11.22	1	26.01	1
RU 0850	0	19.95	1	39.76	1
RU 0284	1	7.40	2	17.65	1
RU 0412	1	17.80	1	43.17	1
RU 0437	0	7.00	2	15.04	1
RU 0438	0	8.90	2	14.21	1
RU 0441	1	3.62	3	8.17	1
RU 0477	1	4.30	2	8.20	1
RU 0487	0	12.00	1	23.80	1
RU 0490	0	5.60	2	11.73	1
RU 0519	0	2.50	3	3.95	2
RU 0551	1	5.10	2	16.44	1
RU 0554	0	13.10	1	29.36	1
RU 0555	1	5.30	2	14.76	1
RU 0576	0	3.23	3	6.65	2
RU 0615	1	11.70	1	26.15	1
RU 0616	0	51.20	0	113.96	0
RU 0828	0	9.50	2	28.86	1
RU 0829	0	25.10	0	73.56	0
RU 0832	0	6.90	2	17.91	1
RU 0839	1	3.80	3	12.68	1
RU 0842	0	1.30	3	3.62	2

IOP Cluster – 1 for active and 0 for inactive compounds; ED_20_, Index, Level – parameters of hypotensive activity.

## 2 Experimental design, materials, and methods

A total of 27 new compounds were synthesized as described previously [6], [7], [8], [9], [10], [11] and tested for IOP lowering effect in ocular normotensive rats. These compounds included twenty 9H-imidazo[1,2-a]benzimidazoles, four 10H-pyrimido[1,2-a]benzimidazoles, two 1H-pyrimido[1,2-a]benzimidazoles and one 1H-imidazo[1,2-a]benzimidazole. All tested compounds were instilled topically in a volume of 0.5 μL.

The animal studies were done in compliance with the ARVO statement for use of animals for vision research and the institutional ethical guidelines. To evaluate 27 imidazobenzimidazoles derivatives for their IOP lowering effect, 3 different concentrations 0.1%, 0.2% and 0.4% were prepared for topical application. Among 27 compounds, 25 were water soluble and these water-soluble compounds were dissolved in 0.25% hydroxypropylmethyl cellulose (HPMC) in distilled water and the solution was filtered using 0.22 μm Millipore filter. HPMC was prepared by measuring 25 mg of HPMC and dissolving in 10 ml of distilled water. To prepare 0.4% concentration, water soluble compounds were weighed to 0.4 mg and dissolved in 1 ml (0.25%) HPMC, then serial dilution was done to obtain 0.2% and 0.1% concentrations. The remaining water insoluble compound was dissolved in 0.1% DMSO in 0.25% HPMC and similarly 3 concentrations of this compound were prepared for topical application.

IOP was measured in the conscious rats using TonoLab (Icare, Finland) rebound tonometer specifically designed for rodents (rat/mouse). Since it is a noncontact tonometer, it does not require use of an anaesthetic agent. The TonoLab was placed right at the centre of the cornea and the distance from the tip of the probe to surface of the cornea was 1–4 mm. For this study, 3 rats were used in each group and the left eye (TE) served as treatment eye while the right eye served as control eye (CE). IOP was measured at 0.5, 1, 1.5, 2, 3, 4, 5 and 6 h post-instillation. Six readings were obtained at each time point and the mean was taken as the final measurement.

Hypotensive activity of tested compounds was evaluated in anesthetized animals (pentobarbital, i.p. 50 mg/kg/bw, JSC Tallinn Pharmaceutical Plant, Estonia) as described previously [12], [13]. Tested compounds were administered in jugular vein. Systemic arterial pressure (SAP) was recorded through carotid artery for 1 h after the administration of the compound using a mercury manometer ПМР-2 (Russian Federation). The measure of hypotensive activity was presented as ED_20_, a concentration (mol/kg) causing maximum SAP to decrease by 20% in 1 h. Additionally, scale was introduced to assess the potency of compounds [14]: index (Ind) of 3 points was applied for ED_20_≤ 4.0 mg/kg, 2 points for ED_20_ = 4.0 ÷ 10.0 mg/kg; 1 point for ED_20_ = 10.0 ÷ 25.0 mg/kg and 0 points for ED_20_> 25.0 mg/kg. Bendazole, (2-(phenylmethyl)-1H-benzimidazole (OJSC Pharmacon Co., St. Petersburg, Russian Federation) was used as a reference drug, with ED_20_ = 18.8 mg/kg = 90.3 μM/kg and Index = 1.

The separation of the studied substances into active and inactive classes was carried out by means of a cluster analysis of 6 indicators of IOP lowering activity by the k-means method using the Statistica 6.0 package [15].

The pharmacophore analysis was performed using the IT Microcosm 7.2 package [14]. First, the chemical structures were translated into descriptors of the QL language [16] using the utilities ActUtil, TranQL2, and MakeData. Then, with the help of the FarmFor module, for each type of QL descriptors, the following were calculated: *P*_a_ – frequency for a class of active compounds; *P*_i_ – frequency for a class of inactive compounds; Pr – significance in hypergeometric test [16]. The QL descriptor was considered a potential pharmacophore of presence of IOP lowering activity if Pr ≤ 0.05 and *P*_a_ > *P*_i_. The QL descriptor was considered a potential pharmacophore of absence of IOP lowering activity if Pr ≤ 0.05 and *P*_i_> *P*_a_.

Neural network simulation of relationship between IOP and hypotensive activities was performed by method of multi-layer perceptron artificial neural networks using the Statistica 6.0 package [15]. Within the framework of the classification model, the architecture of the neural network in the form of a two-layer perceptron was used. As input neurons there were four indicators of hypotensive activity: ED_20_ (mg/kg), Ind, ED_20_ (μM/kg), Lev (the meaning of these parameters is described in [14]). As output neurons, IOP lowering activity indicators were: IOP Cluster (1) – presence of activity, IOP Cluster (0) – absence of activity. Neural networks were constructed in automatic mode ANN, random sampling 80% for training and 20% for testing. The following parameters of the neural network simulation were set: 1) multilayer perceptron MLP; 2) the minimum number of hidden neurons 3; 3) the maximum number of hidden neurons 10; 4) number of trained networks 1000; 5) number of selected good networks 50; 5) types of activation functions for hidden and output neurons Identity, Logistic, Tanh, Exponential, Sine, Softmax; 7) other parameters were accepted by default. After training, from the 50 automatically selected good networks, based on the accuracy of training and testing, the best network was selected. The statistical correspondence of the experimental and calculated estimates of IOP lowering activity was determined using Statistica 6.0 package [15] by means of nonparametric Spearman correlation coefficient.

## Footnotes

Appendix A Supplementary data associated with this article can be found in the online version at doi:10.1016/j.dib.2018.02.067.
