# Supplementary material for: Relationship between intraocular pressure lowering effect and chemical structure of imidazo[1,2-a]benzimidazole and pyrimido[1,2-a]benzimidazole derivatives
Source: Data Brief. 2018 Mar 6;18:340–7. doi: 10.1016/j.dib.2018.02.067 (PMC5995800; doi:10.1016/j.dib.2018.02.067)
Supplement: Supplementary file 1 — Supplementary material [file mmc1.docx]

**Conflict of interest**

There is no conflict of interest. All authors have substantially contributed to conception, designing, drafting the article and in final approval of the manuscript version to be submitted. All authors have jointly decided to designate Assoc Prof Dr Igor Iezhitsa to be responsible for taking decision regarding the presence of authors and the order of their presence in the manuscript. Assoc Prof Dr Igor Iezhitsa has also been selected by all authors to be responsible for all future communication with the journal regarding this manuscript.
